# A case report of surgical resection treatment for complete remission after chemotherapy for advanced pancreatic cancer

**DOI:** 10.3389/fonc.2023.1155233

**Published:** 2023-05-25

**Authors:** Zhongyan Zhang, Hongfeng Lin, Hehe Li, Xin Wang

**Affiliations:** ^1^ Department of Hepatobiliary Surgery, Weifang People's Hospital, Weifang, China; ^2^ Department of Geriatrics, Weifang People's Hospital, Weifang, China

**Keywords:** advanced pancreatic cancer, gemcitabine, chemotherapy, surgical resection, case report, nab-paclitaxel (nab-P)

## Abstract

Pancreatic cancer is a common type of cancer that is treated using surgery or chemotherapy. However, for patients who cannot have surgery, the treatment options are limited and have a low success rate. We report a case of a patient with locally advanced pancreatic cancer who was unable to have surgery due to a tumor that had invaded the coeliac axis and portal vein. However, after receiving chemotherapy with gemcitabine plus nab-paclitaxel(GEM-NabP), the patient achieved complete remission, and a PET-CT scan confirmed that the tumor had disappeared. Eventually, the patient underwent radical surgery with distal pancreatectomy with splenectomy, and the treatment was successful. This case is rare, and there are few reports of complete remission after chemotherapy for pancreatic cancer. This article reviews the relevant literature and guides future clinical practice

## Introduction

Pancreatic cancer is one of the most commonly seen malignant tumors in clinical practice, with a high mortality rate (1). Since the early clinical symptoms of pancreatic cancer are not obvious, most patients are already in the advanced stage when the diagnosis is confirmed. Due to the advanced stage of cancer, surgical outcomes are poor and the cancer is often declared inoperable. Currently, the main treatment methods for pancreatic cancer are surgical resection, chemotherapy, immunotherapy, radiation therapy, and local therapies such as radiofrequency ablation and irreversible electroporation (2). Surgical treatment is still recognized as the only potential cure for pancreatic cancer, with a 5-year survival rate of 10% to 25% for patients who undergo surgical resection. However, in patients with resectable pancreatic cancer, it is essential to ensure that the surgical margins are negative (R0) to extend the patient’s survival. For advanced pancreatic cancer patients, surgery is often palliative because it is difficult to achieve a curative (R0) resection effect (3). In recent years, with the widespread application of neoadjuvant chemotherapy for pancreatic cancer, patients in the advanced stage have had the opportunity for curative surgery (4). However, there is currently a lack of sufficient clinical research evidence. This article is based on a clinical case of a patient with locally advanced pancreatic cancer who achieved complete remission after conversion therapy and underwent curative surgery. It also reviews relevant literature in the hope of helping treatment options for locally advanced pancreatic cancer patients.

## Case description

**Figure 1:**
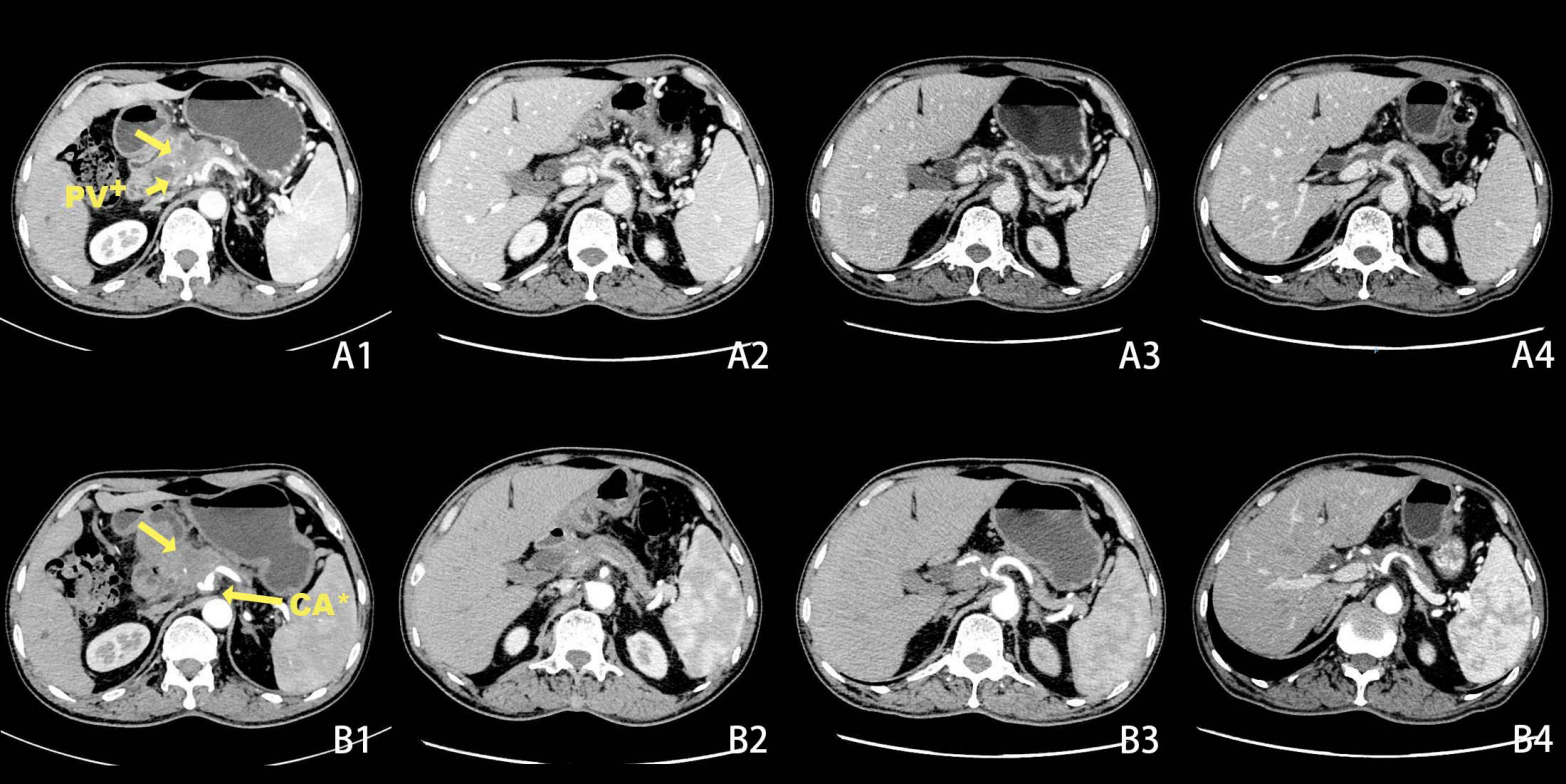
Changes in tumor size before and after chemotherapy on CT scans + Portal Vein, * Coeliac Axis. Before chemotherapy CT scan of the abdomen shows a lesion in the pancreatic neck with portal vein and coeliac axis invasion **(A1, B1)**. After 2 cycles of chemotherapy, the tumor had significantly decreased in size **(A2, B2)**. After 5 cycles of chemotherapy **(A3, B3)**. After 6 cycles of chemotherapy**(A4, B4)**.

**Figure 2:**
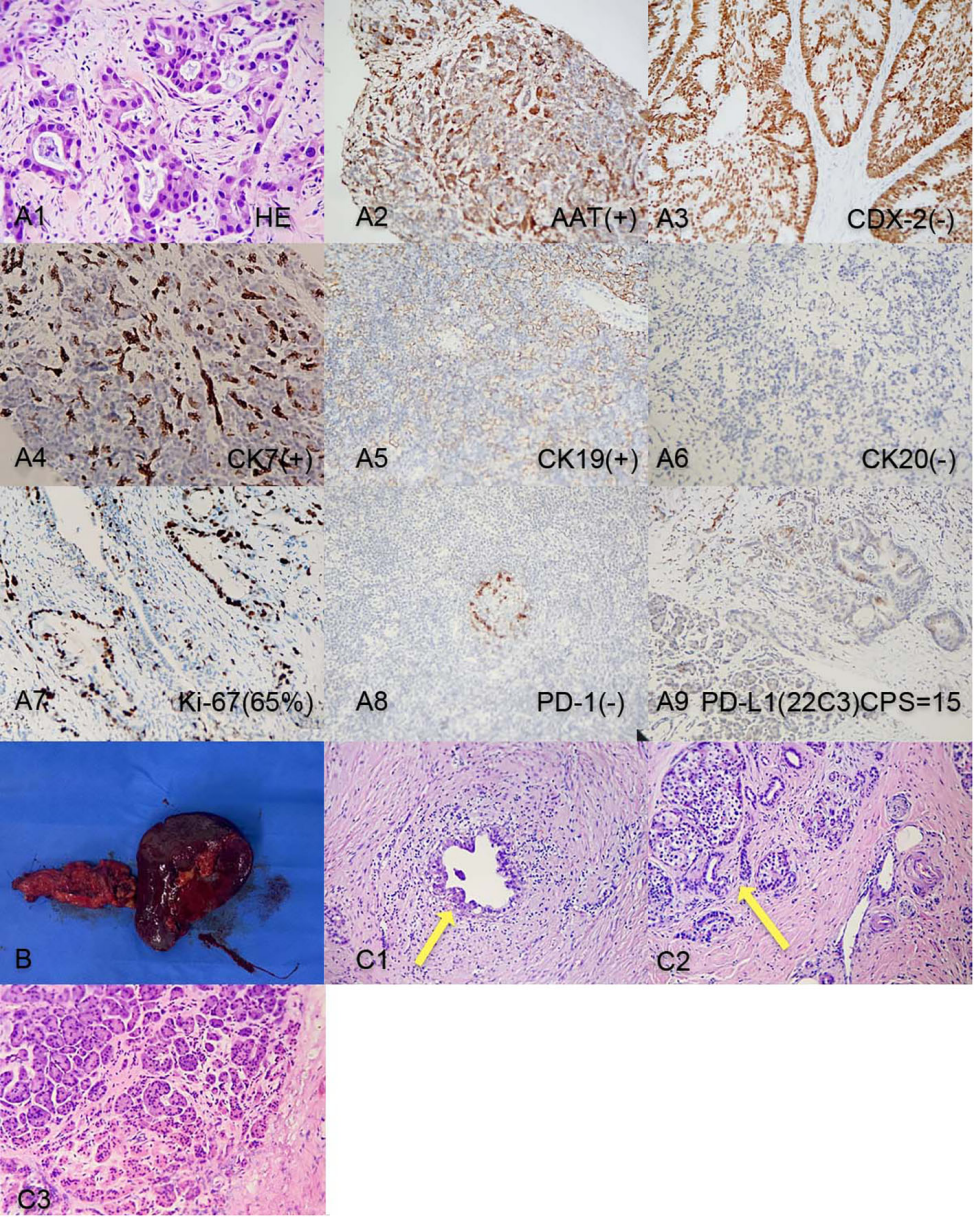
**(A1-A9)** Pancreatic tumor biopsy pathology results before chemotherapy. **(B)**. Post-surgery tissue of the tail of the pancreas and spleen; **(C1)** Post-surgery pathology: Residual pancreatic ductal adenocarcinoma cells; **(C2)** Post-surgery pathology: Pancreatic islet cell hyperplasia and ductal dilation; **(C3)** Intraoperative pathology result: no obvious malignant cells on the cut end of the pancreas.

A 50-year-old male patient presented to the clinic in June 2022 with “upper abdominal pain for 1 week”. Physical examination revealed upper abdominal tenderness, no rebound pain, no palpable abdominal masses, no jaundice on the skin and mucous membranes, and no obvious enlargement of the supraclavicular lymph nodes. Laboratory tests showed (on June 4, 2022) a significantly elevated CA19-9 level (394.4 U/mL, normal range 0-34 U/mL), while total bilirubin, CA125, AFP, and CEA were all within normal ranges. A chest and abdomen flat scan and contrast-enhanced CT (**Figures 1A1, B1**) on June 4, 2022, revealed a low-density area with unclear boundaries at the junction of the neck and body of the pancreas, mild enhancement on enhanced scans; dilation of the main pancreatic duct and involvement of the bile duct pancreatic segment, with thickening and obvious enhancement of the wall. The pancreatic neck and surrounding areas have been affected, including the bile duct system, pancreatic ducts, portal vein, splenic vein, and hepatic artery (AJCC T3N1M0). The whole body bone scan shows no obvious bone metastases. On June 9, 2022, a CT-guided pancreatic biopsy showed moderately differentiated pancreatic adenocarcinoma. Immunohistochemistry shows that tumor tissues are positive for CK7, CK19, and AAT, but negative for CK20, and CDX2. PD-1 is negative and PD-L1 expression is 15%. The Ki-67 index is about 65%. According to the WHO classification, our hospital’s pathologists diagnosed it as a primary moderately differentiated pancreatic ductal adenocarcinoma(PDAC) (**Figure 2A1–A9**).

**Figure 3:**
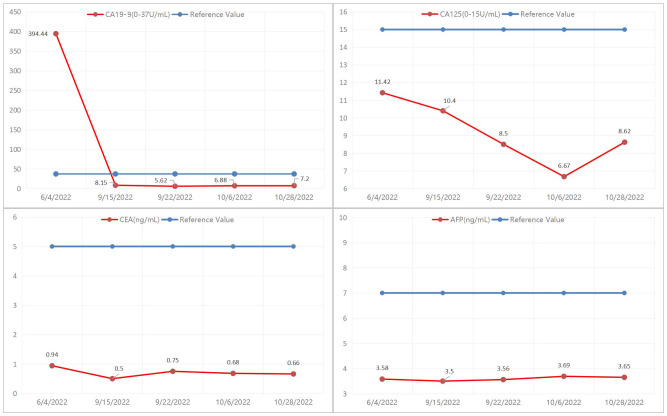
Trend of change in tumor marker expression level before and after chemotherapy.

The patient is a 50-year-old male with pancreatic adenocarcinoma(AJCC T3N1M0) that invades the coeliac axis and portal vein. He underwent GEM-NabP chemotherapy for 6 cycles. After 6 cycles of chemotherapy, repeat abdominal CT showed progressive tumor shrinkage (**Figures 1A1–B4**), and tumor marker expression levels gradually decreased (**Figure 3**). On October 31, 2022, a PET/CT was performed. PET/CT scan showed a slightly low density in the pancreatic neck and body junction after pancreatic cancer treatment. No obvious increase in radioactive distribution was observed in the lymph nodes around the pancreas. The treatment effect was evaluated as complete remission.

After the last chemotherapy treatment, the patient’s blood test, liver and kidney function showed no obvious abnormalities. The patient’s HGB level was 74g/L, after a transfusion of 3.5U of packed red blood cells, the HGB level increased to 104g/L, correcting the anemia. On November 7, 2022, the patient underwent a pancreatic neck tumor and distal pancreatectomy with splenectomy. The surgery lasted for 395 minutes, with an estimated blood loss of 600ml, and a transfusion of 3.0U of packed red blood cells and 450ml of plasma. The surgery was successful (**Figure 2B**). The pathology report after the surgery (**Figures 2C1–3**) showed a small amount of residual adenocarcinoma at the original tumor site with islet cell proliferation, ductal dilation, no obvious intratumoral thrombosis or nerve invasion, and no lymph node metastasis. According to the College of American Pathologists(CAP) grading system for tumor regression, this case belongs to grade 1(near-complete response with single cancer cells or rare small groups of cancer cells) (5).

## Discussion

Pancreatic cancer is one of the worst prognoses of all cancers, 15% of pancreatic cancer patients have the resectable disease. 35% have locally advanced pancreatic cancer(LAPC) and 50% have metastatic disease (MPA). Unresectable pancreatic cancer includes LAPC and MPA. LAPC mainly refers to the primary tumor that cannot typically be removed safely because reconstruction of major vessels is seemingly not possible. MPA spreads to distant organs or non-regional lymph nodes (5). Currently, curative surgery (R0 resection) is still the only treatment that can potentially achieve a clinical cure for pancreatic cancer patients (6). However, due to the advanced stage of the disease when patients seek treatment, pancreatic cancer patients are very likely to invade surrounding organs and blood vessels, resulting in a low R0 resection rate (7).

For patients who have LAPC, chemotherapy is often used as a treatment option. Before chemotherapy, a pathological diagnosis is usually obtained. EUS-guided biopsy represents the gold standard for pathological diagnosis in pancreatic cancer patients. A multicenter, randomized, crossover trial comparing the wet-suction versus slow-pull technique for EUS-guided fine-needle biopsy showed that the wet-suction technique had a significantly higher diagnostic yield and fewer needle passes compared to the slow-pull technique (8). It is important to note that while CT-guided biopsy was used in this case, EUS-guided biopsy should be considered the preferred method for diagnosis in pancreatic cancer patients.

Evaluation of treatment response includes RECIST criteria and CA19-9 levels after chemotherapy (5). Studies have shown that the expression level of CA19-9 is inversely related to the prognosis of patients, the higher the expression level of CA19-9, the worse the prognosis of patients (9). Previous studies have shown that the decline of CA19-9 in LAPC patients after neoadjuvant therapy is more than 50%, which is closely related to a better prognosis. In this case, the patient’s CA19-9 expression level was 394.4 U/mL before neoadjuvant therapy, and after 6 cycles of GEM-NabP chemotherapy, the CA19-9 expression level decreased to 7.2 U/mL. Our hospital’s PET/CT showed that the tumor size decreased, the lesion metabolism decreased, and the CA19-9 decreased more than 50% of the initial value, considering the patient’s disease remission after chemotherapy, and then underwent pancreatic cancer radical surgery. Although preoperative PET/CT showed no tumor activity, postoperative pathology confirmed that there were still small amounts of adenocarcinoma residues. Therefore, it can be suggested that PET/CT is not accurate when the tumor cell content is very low.

With the popularization of multidisciplinary treatment concepts, neoadjuvant therapy has gradually become prominent in the treatment of pancreatic cancer. Neoadjuvant therapy aims to downstaging the tumor and eliminate micrometastases, thereby improving the R0 resection rate and reducing recurrence and metastasis. Previous reports have shown that the R0 resection rate of pancreatic cancer patients after neoadjuvant chemotherapy can reach 81.8%. In our hospital, the patient, in this case achieved partial remission after 6 cycles of GEM-NabP chemotherapy and underwent radical surgery, achieving R0 resection. Therefore, late-stage pancreatic cancer patients are more likely to achieve R0 resection after neoadjuvant chemotherapy. Currently, the main neoadjuvant chemotherapy regimens for advanced pancreatic cancer are FOLFIRINOX (oxaliplatin + irinotecan + leucovorin + 5-fluorouracil) and GEM-NabP (10). Studies have shown that neoadjuvant chemotherapy can effectively improve the pathology of pancreatic cancer, reduce the invasion of lymphatic vessels in patients, increase R0 resection rates, and improve overall survival (**Table 1**, (11)). However, for the treatment regimens of LAPC, there is still a lack of a large number of clinical experimental studies to prove.

**Table 1:** Recent trials on neoadjuvant chemotherapy, resectability, and outcomes.

Study	Design	Local stage	Chemotherapy	n	Resectability	R0 rate	Median os (months)
McKenzie 2013	Phase 2 trial	Res	Gem/nP	25	80.0%	95%	NA
O'Reilly 2014	Phase 2 trial	Res	Gem/oxaliplatin	38	71.0%	74%	27.2
Sliesoraitis 2014	Phase 2 trial	Res/BR	Gem/nP VS surgery	32 (10 VS 22)	80% VS 100%	60% VS 77%	NA
lelpo 2016	Phase2 trial	Res/BR	Gem/nP	25	68.0%	100%	21
Katz 2016	Phase 1/2 trial	BR	mFOLFIRINOX	22	68.0%	93%	21.7
Okada 2017	Phase 1 trial	BR	Gem/nP	10	80.0%	70%	NA
Tsai 2018	Phase 2 trial	Res/BR	5-FU or gemcitabine-based chemotherapy,depending on molecular profiling	130	82.0%	81%	38
Reni 2018	Phase 2/3 trial	BR/LA	Gem/nP VS Gem/nP/cis/cap	54 (28 VS 26)	32% VS 31%		NA
Murphy 2018	Phase2 trial	BR	FOLFIRINOX+CRT	48	66.6%	97%	37.7
De Marsh 2018	Phase2 trial	Res	FOLFIRINOX	21	81.0%	94%	34
Wei 2019	Phase2 trial	Res	Gem/erlotinib	114	73.0%	81%	21.3
Barbour 2020	Phase2 trial	Res	Gem/nP	42	71.4%	86%	23.5
Sohal 2020	Phase2 trial	Res	FOLFIRINOX VS Gem/nP	55 VS 47	73%VS 70%	85%VS 85%	22.4 VS 23.6

Gem, gemcitabine; nP, nab-paclitaxel; CRT, chemoradiation therapy; OS, overall survival; Res, resectable; BR, borderline resectable.

## Conclusion

In summary, for patients with locally advanced pancreatic cancer who have responded to chemotherapy further surgery is feasible. However, long-term follow-up is still necessary to evaluate the patient’s long-term outcomes and survival. Large-scale trials are also needed to validate these conclusions.

## Data availability statement

The raw data supporting the conclusions of this article will be made available by the authors, without undue reservation.

## Ethics statement

Written informed consent was obtained from the individual(s) for the publication of any potentially identifiable images or data included in this article.

## Author contributions

ZZ: guarantees the integrity of the entire case and edited the manuscript. HL and XW: performed the literature research, data analysis, and text proofreading. All authors contributed to the article and approved the submitted version.
